# Use of complementary medicine among patients with allergic rhinitis: an Italian nationwide survey

**DOI:** 10.1186/s12948-019-0107-1

**Published:** 2019-02-13

**Authors:** G. Bonizzoni, M. Caminati, E. Ridolo, M. Landi, M. T. Ventura, C. Lombardi, G. Senna, M. Crivellaro, F. Gani

**Affiliations:** 1 Allergy Service AOU San Luigi Hospital Orbassano, Turin, Italy; 2 Asthma Center and Allergy Unit and Department of Medicine, Verona University Hospital, Piazzale Scuro, 37134 Verona, Italy; 3 Experimental and Clinical Medicine, University of Parma, Parma, Italy; 4 National Health Care System, Turin, Italy; 5 Department of Interdisciplinary Medicine, University of Bari, Bari, Italy; 6 Department Unit of Allergology and Respiratory Diseases, Fondazione Poliambulanza Hospital Institute, Brescia, Italy; 7 Department of Cardiac, Thoracic and Vascular Sciences, University of Padua, Padua, Italy

**Keywords:** Complementary medicine, Allergic rhinitis

## Abstract

**Background:**

A growing use of complementary alternative medicine (CAM) has been found in Europe as well in Italy for chronic diseases, including the allergic rhinitis. The study aims at investigating the prevalence and the pattern of use of CAM amongst patient with allergic rhinitis.

**Methods:**

A 12-item questionnaire was developed by a panel of experts and administered to patients with moderate/severe allergic rhinitis consecutively referring during the study time-frame to seven allergy clinics placed all around Italy. The items covered several topics including reason for choosing CAM, its clinical efficacy, schedule of treatment, costs, type of therapy.

**Results:**

Overall 359 questionnaires were analysed. 20% of patients declared CAM use. A significant correlation between the use of CAM and female sex (p < 0.01) and with a higher level of education (p < 0.01) was observed. CAM users were adults (36% in the range between 20 and 40 years and 32% between 41 and 60 years). Youngsters (< 20 years) (7%) and elderly (> 60) (25%) less frequently used CAM.The most used type of CAM was homoeopathy (77% of patients). 60% of users would recommend CAM despite a poor clinical efficacy according to 67% of them.

**Conclusions:**

Although no evidence supports CAM efficacy and safety, the number of patients who relies on it is not negligible. As allergic rhinitis is not a trivial disease, the use of CAM as the only treatment for it should be discouraged at any level, but by general practitioner and specialist in particular.

## Background

A growing use of complementary alternative medicine (CAM) has been found in Europe as well in Italy for chronic diseases, including the allergic rhinitis [1–3]. Allergic rhinitis is a very common disease; its prevalence in Italy is more than 20% [4]. According to a survey only 48% of patients suffering from rhinitis have seen a medical doctor in the last year and 26% of them used homeopathic therapy or are completely untreated: the cost of allergy medication is the reason for avoiding any treatment in 40% of cases [5]. In a recent Italian survey most of investigated patients (68%) received the prescription of the first therapy in the GP setting, whereas self diagnosis and self treatment were the first choice in the remaining subjects, who looked for advices from pharmacists, internet, magazines, friends and relatives [6]. More than 50% of patients with allergic rhinitis used multiple therapies for their disease, but 40% of them were not satisfied (6). CAM is wildly used as an alternative or in conjunction with traditional treatment. In the present study we aimed at investigating the prevalence and the pattern of use of CAM among patient with allergic rhinitis referring to an allergy clinic.

## Methods

A 12-item questionnaire was developed by a panel of experts and administered to patients with moderate to severe allergic rhinitis, according to ARIA classification [7], consecutively referring to seven allergy clinics placed all around Italy, during the study time-frame (30th May to 31 October 2016). It was intended to be a questionnaire for self-compilation. The items covered several topics the following topics: reason for choosing CAM, its clinical efficacy, schedule of treatment, costs, type of therapy. Demographic data associated with information concerning school education were also collected. Statistical analysis was performed for comparing CAM users with those who had no experience of such methods. Chi squared test was used for the analysis.

## Results

Overall 359 consenting adult respondents were enrolled. The reported prevalence of CAM use was 20% (70 patients). The main findings of the survey are summarized in Tables 1 and 2. A significant correlation between the use of CAM and female sex (p < 0.01) and with a higher level of education (p < 0.01) was observed. CAM users were adults (36% in the range between 20 and 40 years and 32% between 41 and 60 years). Youngsters (< 20 years) (7%) and elderly (> 60) (25%) less frequently used CAM. The most common type of CAM was homoeopathy (77% of patients). Among the CAM users, 67% reported a substantial lack of clinical efficacy, but 61% of them would recommend the treatment. The most common reason for choosing it was that it is a natural treatment; a further reason was the fear of side effects related to traditional medicine.

**Table 1:** Demographic data of sample population

Patients	Total	N. CAM users (%)	N. CAM non users (%)	p value
Sex				
Males	152	18 (25)	134 (25)	
Females	207	53 (75)	53 (75)	< 0.01
Education				
Elementary	43	3 (4)	40 (14)	< 0.05
Middle school	82	13 (19)	69 (24)	
High school	168	30 (42)	138 (48)	
University	66	25 (35)	41 (14)	< 0.01
Age				
< 20	25	4 (7)	21 (7)	> 0.05
20–40	128	30 (36)	98 (34)	
41–60	116	26 (32)	90 (31)	
> 60	90	11 (25)	79 (28)

**Table 2:** Pattern of use in CAM users

	Number pts (%)
Type of therapy^a^	
Homoeopathy	55 (77)
Herbal remedies	28 (39)
Acupuncture	8 (11)
Other	8 (11)
Treatment duration	
< 6 months	20 (28)
> 6 months	52 (72)
Schedule of treatment	
Regular	43 (60)
On demand	28 (40)
Clinical efficacy	
Yes	24 (33)
No	47 (67)
Cost/month	
< 50 Euro	32 (45)
> 50 euro	39 (55)
Reason for choice^a^	
Natural	40 (56)
Fear of side effects of traditional Medicine	39 (55)
Dissatisfaction with traditional medicine	13 (18)
Other	21 (30)
Providers of information^a^	
GP	26 (37)
TV or newspaper, websites	31 (40)
Family and friends	34 (48)
Other	6 (8)
Recommendation for CAM use	
Yes	43 (61)
No	28 (39)

^a^More than one choice for each patient

Mass media (40%) and family or friends (48%) were the major source of information about CAM. Of notice 37% of CAM users declared they received information form their General Practitioners (GPs).

## Discussion

This survey showed a fairly large use of CAM among patients with allergic rhinitis, mainly adults, though with poor benefits, as reported by 67% of respondents. Homoeopathy was the main form of CAM used, followed by herbal remedies. Despite the ARIA guideline do not suggest the use of CAM [8], GPs prescribed or recommended CAM to their patients in 37% of cases. Other important providers of information were newspapers and the web. This finding might account for the prevalence of CAM users among young adults, who are more familiar with these means of communication. CAM costs are comparable or even higher than traditional therapies; patients with high education level are more frequently “CAM consumers”, perhaps because they can better afford its costs. Though CAM is pricey and patients pay out of pocket its costs, patients prefer to follow the treatment on regular basis. However, no data about the adherence to these treatments are available.

The more frequent reason for the choice was the fear of potential side effects related to traditional medicine. However, despite the common belief that CAM is completely safe, there is a risk of toxicity, malignancies, mechanical injuries and drug interaction [9]. Recently also severe allergic reaction has been reported [10].

The survey results highlight two major pitfalls in the management of allergic rhinitis. First, patients do not refer to their GP or to the specialist when they suffer from nasal symptoms. Second, strictly connected with the first one, there is a substantial lack of knowledge about the treatment options for nasal symptoms, their potential benefits and their side effects. One potential explanation is that the nasal symptoms are considered somehow trivial, so they not deserve a serious assessment according to the patients, as previously described [6, 11]. As allergic rhinitis is not a trivial disease, the use of CAM as the only treatment for it should be discouraged at any level, but by general practitioner and specialist in particular. Also, as pharmacies are often the first line of referral for the patients suffering from allergic rhinitis [6, 11], they should be more extensively involved in shared educational programmes so that they support doctor in providing correct information about nasal symptoms treatment options and promoting medical referral for the best assessment.
